# Preventing action slows down performance in perceptual judgment

**DOI:** 10.1007/s00221-020-05948-y

**Published:** 2020-10-13

**Authors:** D. Quarona, M. Raffuzzi, M. Costantini, C. Sinigaglia

**Affiliations:** 1 Department of Philosophy, University of Milan, Milan, Italy; 2 Cognition in Action (CIA) Unit, PHILAB, University of Milan, Milan, Italy; 3 Department of Psychological, Health, and Territorial Sciences, “G. D’Annunzio” University of Chieti-Pescara, Chieti, Italy; 4 Institute for Advanced Biomedical Technologies—ITAB, University “G. D’Annunzio”, Chieti, Italy

**Keywords:** Action and perception, Visuo-motor aftereffect, Grasping, Canonical neurons

## Abstract

Action and vision are known to be tightly coupled with each other. In a previous study, we found that repeatedly grasping an object without any visual feedback might result in a perceptual aftereffect when the object was visually presented in the context of a perceptual judgement task. In this study, we explored whether and how such an effect could be modulated by presenting the object behind a transparent barrier. Our conjecture was that if perceptual judgment relies, in part at least, on the same processes and representations as those involved in action, then one should expect to find a slowdown in judgment performance when the target object looks to be out of reach. And this was what we actually found. This indicates that not only acting upon an object but also being prevented from acting upon it can affect how the object is perceptually judged.

## Introduction

Action and vision are known to be tightly coupled with each other. Many studies show that visual perception might affect action. For instance, when visually presented with various objects, participants are faster in performing a compatible action, even when the action does not pertain to the viewed object (Costantini, Ambrosini, Tieri, Sinigaglia, and Committeri 2010; Ellis and Tucker 2000; Tucker and Ellis 1998, 2001,2004). Some studies indicate that the other way around could be true as well, with action affecting visual perception (Craighero, Fadiga, Rizzolatti and Umiltà 1999; Hecht, Vogt and Prinz 2001; Cardoso-Leite, Mamassian, Schütz-Bosbach, Waszak 2010; Brockmole, Davoli, Abrams, and Witt 2013; Chan, Peterson, Barense, and Pratt 2013; Davoli and Tseng 2015; Gozli, West, and Pratt 2012).

In a previous series of experiments, we explored the action–vision coupling by investigating whether and how repeatedly performing a reach-to-grasp action might influence the perceptual judgement on a relevant visual feature of an object (i.e. a mug), such as its shape (its being handled or not). We found that repeated grasping actions produced a perceptual after-effect, with participants being slower in detecting the visually presented handled mugs when their handles were oriented congruently with the grasping hand. Interestingly, the same effect did not occur when participants were asked to detect the visually presented mugs after repeatedly performing reach-to-touch actions (Costantini, Tommasi, and Sinigaglia 2019).

This finding suggests that perceptual judgments of visual object features may rely on motor processes and representations typically recruited when acting. This raises naturally a further question. Could altering the visual presentation of object features by manipulating specific action-related cues affect perceptual judgment? The main aim of the present study is to answer this question. To this end, we introduce a transparent barrier in front of the object, thereby making it appear to be out of reach, that is, as something that could not be acted upon.

There is evidence that presenting an object, such as a handled mug, beyond a near transparent barrier impacts on how its features (e.g. handle orientation) are processed motorically. In a behavioural study, we instructed participants to pantomime a reach-to-grasp action, with either the right or the left hand, on the presentation of a task-irrelevant go-signal. The go-signal was a 3D scene in which a mug, placed on a table, had the handle oriented toward the left or right, thus being congruent or incongruent with the pantomimed action. In half of the trials, the mug was located in front of a transparent plexiglas panel, whereas in the other half, it was located beyond the same panel, thus appearing to be out of reach. The results showed that participants were faster in responding with their reach-to-grasp pantomime when the handle of the mug was oriented congruently with the hand to be used, provided that the mug was presented in front of (but not behind) the plexiglas (Costantini et al. 2010).

Here, we took advantage of the same strategy by investigating whether and how the insertion of a transparent barrier making a visually presented object appear to participants to be out of reach would impact on their performance in perceptual judgment. As in Costantini et al. (Costantini et al. 2019), we asked participants to judge whether a mug was handled or not after motor training. This motor training consisted in repeatedly reaching for and grasping a handled mug without seeing their hand and without seeing the target object. When judging, some participants were visually presented with the mug behind a transparent barrier. The other participants performed the judgment perceptual task without any barrier. In a control condition, both judgment perceptual tasks were performed after an alternative form of motor training which consisted in repeatedly reaching for and touching the mug with the hand closed in a fist. As in the other motor training, neither the moving hand nor the object target was visible. If perceptual judgment relies, in part at least, on motor processes and representations of the same kind as those typically involved in action execution, then one should expect that making the target object appear as something that could not be acted upon would selectively impact on performance in making perceptual judgements. In particular, one should expect that presenting participants with a mug beyond a transparent barrier should affect their way of judging its being handled when, and only when, their motor training required a reach-to-grasp action.

## Materials and methods

### Participants

Eighty participants were recruited and randomly assigned to each of four groups: Grasp-without-barrier (mean age ± s.d. = 22.40 ± 2.32), Touch-without-barrier (mean age ± s.d. = 22.35 ± 2.43), Grasp-with-barrier (mean age ± s.d. = 22.60 ± 3.57), and Touch-with-barrier (mean age ± s.d. = 22.30 ± 3.48; F (3, 1) = 0.495, *p* = 0.736), respectively. Participants were all right-handed, with normal or corrected-to-normal vision, and with no history of either psychiatric or neurological disorders as self-reported. The study was approved by the local ethics committee and carried out in accordance with the principles of the revised Helsinki Declaration (World Medical Association General Assembly, 2008). Written informed consent was obtained from all the participants.

### Stimuli and procedure

**Fig. 1:**
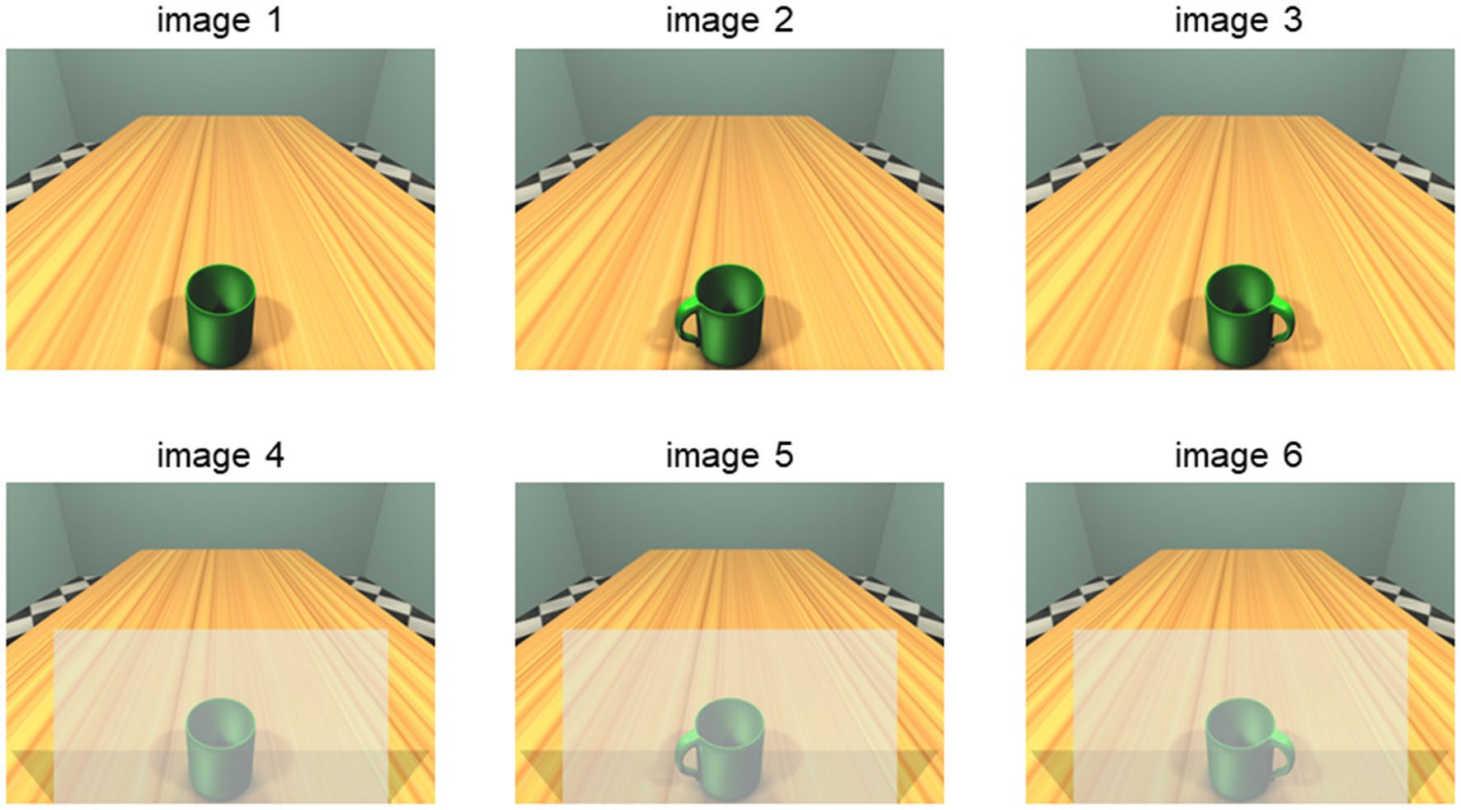
Experimental stimuli. Images 1, 2 and 3 depict a mug without handle, a left-handled and a right-handled mug, respectively. Images, 4, 5, and 6 depict each of the three mugs behind a semitransparent barrier

Experimental stimuli consisted of six pictures (1920 × 1040) representing a 3D room with a table and a mug on it, similar to the stimuli used in our previous study (Costantini et al. 2019). The mug could be either handled or not. In the former case, the handle was either right- or left-oriented. In half of the stimuli, a semitransparent barrier was placed before the mug (see Fig. 1). Hence, we had three stimuli showing the mug, either handled or not; and three stimuli showing the mug, either handled or not, with the semitransparent barrier.

The experiment took place in a dimly lit room. The experiment consisted of motor training followed by a perceptual judgment task. In the motor training, participants of the Grasp-without-barrier and Grasp-with-barrier groups were instructed to repeatedly reach for and grasp with their right hand, for 3 min, the handle of a real mug positioned 25 cm from their body midline, without picking it up. The grasping hand always started from the same instructed position, with thumb and index forming a pinch grip. Participants were asked to synchronize every movement to a metronome (40 bpm), so that each movement lasted approximately 1500 ms. Each participant performed 120 movements. A black box was used to prevent participants from seeing their own movement or the mug, and back tissue covered participants’ right hand throughout the motor training. Participants assigned to both the Touch-without-barrier and Touch-with-barrier groups were given different motor training: rather than reaching for and grasping the handle of the mug, participants assigned to these groups were asked to close their hands in a fist and repeatedly reach for and touch the mug with their knuckles.

**Fig. 2:**
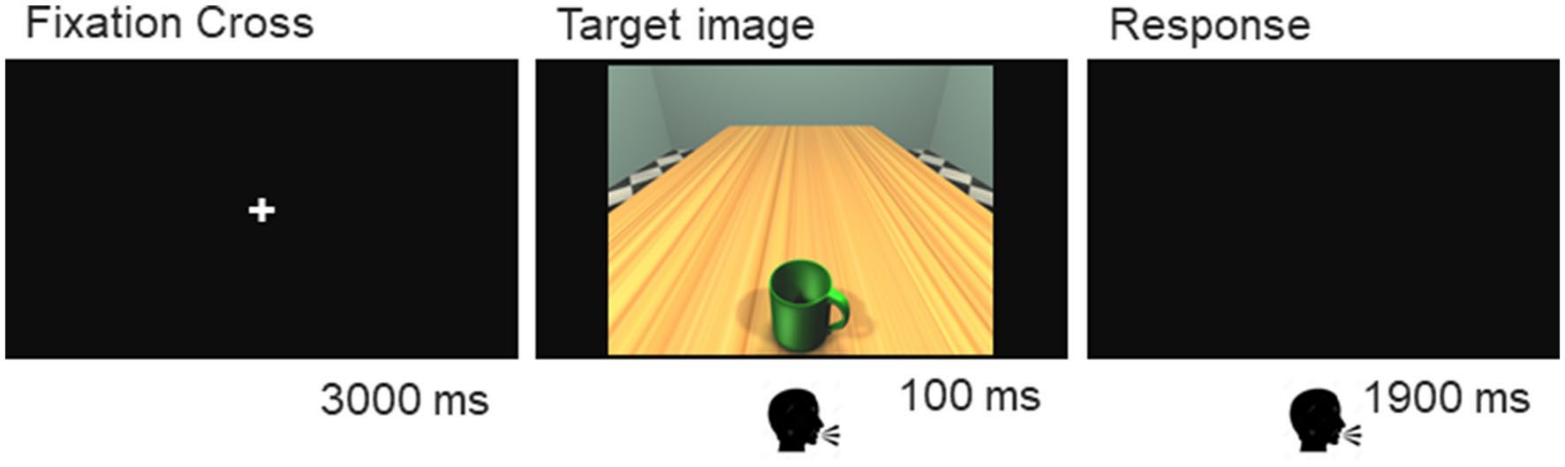
Trial Structure. A fixation cross lasting 3000 ms was followed by the target image, showed for 100 ms. After that a response slide was displayed for 1900 ms

In the perceptual judgment task, participants sat in front of a 21-inch computer screen (1920 × 1080 pixels; refresh rate = 60 Hz) at a viewing distance of 60 cm. They were asked to detect the presence or the absence of the handle by saying into a microphone “sì” (yes) when the handle was present and “no” otherwise. Each trial started with a fixation cross lasting 3000 ms, followed by one of the stimuli lasting 100 ms. After the stimulus, a response slide consisting of a black screen was displayed for 1900 ms (see Fig. 2). Accuracy and reaction times were collected. Reaction times were recorded from the presentation of the stimulus; hence, participants had 2000 ms to provide their response. The perceptual task lasted approximately 6 min. Stimuli, timing and randomization procedure was controlled by E-Prime Software 3.0 (Psychology Software Tools).

**Fig. 3:**
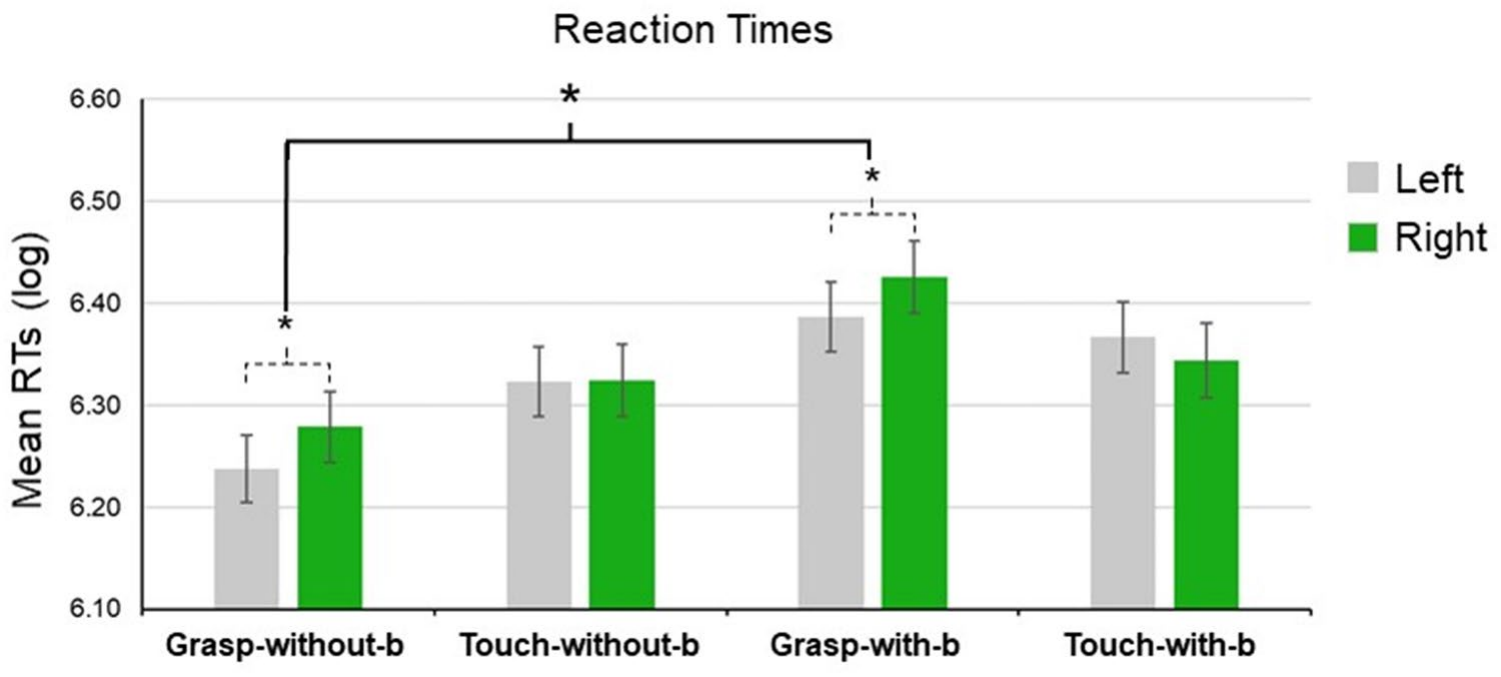
Mean Log-RT for right-handled and left-handled stimuli. Participants of the Grasp-without-barrier group performed a grasping motor training and were exposed to target images without the semitransparent barrier; participants of the Touch-without-barrier group performed a touching motor training and were exposed to target images without the semitransparent barrier; participants of the Grasp-with-barrier group performed a grasping motor training and were exposed to target images with the semitransparent barrier; participants of the Touch-with-barrier group performed a touching motor training and were exposed to target images with the semitransparent barrier. Straight line indicates the significant post-hoc of the main effect of group; dashed lines indicate significant post-hoc of the group by handle interaction. Error bars indicate standard errors; * indicates *p* < .05

The perceptual judgment task was composed of two equal blocks of 40 trials. During each block, a picture of the mug with no handle was randomly presented for 20 trials; meanwhile, a picture of the mug with the handle on the left and a picture of the mug with the handle on the right were randomly presented for 10 trials each. The experimental procedure was the same for all the groups, except for the fact that the participants assigned to both Grasp-with-barrier and Touch-with-barrier groups were presented with the mug behind a semitransparent barrier. Participants of the both Grasp-without-barrier and Touch-without-barrier groups were presented with the mug only.

### Data analysis

Trials with the no-handle mug were excluded from the analysis because “no” responses cannot be compared to “yes” responses. Indeed, saying ‘yes’ and ‘no’ (‘sì’ and ‘no’ in Italian) is different in terms of labial articulation and, consequently, in their acoustic wave forms. Since vocal reaction times are computed on the base of words’ wave forms, the criteria (i.e., when word waves start to be different from noise sound) by which we select vocal initiation of ‘yes’ and ‘no’ words are very different and, therefore, not directly comparable. Furthermore, in visual search tasks, reaction time (RT) is a roughly linear function of set size. Note that RTs will increase at a rate of approximately 20–30 ms/item for target-present trials and 40–60 ms/item for target-absent trials (Wolfe 1998, 2020). RTs are dramatically slower in the target-absent trials and this makes them not directly comparable to RTs in the target-present trials.

In line with previous studies on perceptual adaptation (Cattaneo, Sandrini, and Schwarzbach 2010; Costantini, et al. 2010), we considered only the first 40 trials. Reaction times (RTs) were log-transformed. RTs of “yes” responses deviating more than ± 2 s.d. at the subject level were treated as outliers and not considered for analysis (outliers < 1.75% for group 1, outliers < 1.25% for group 2, outliers < 1.95% for group 3, outliers < 1.20% for group 4). Participants deviating more than ± 2 s.d. from the group performance for either reaction times or accuracy were not included in the analysis. In total, eight participants were discarded (1, 2, 2 and 3 for each of the four groups, respectively).

RTs were submitted to a 2 × 4 mixed analysis of variance (ANOVA) with handle orientation (left vs. right) as a within-subject factor and group (1 vs. 2 vs. 3 vs. 4) as a between-subject factor. For all the statistical tests, the alpha level was set to 0.05. All multiple comparisons were Bonferroni corrected.

## Results

Accuracy was equal or larger than 97% in all the groups and conditions, thus not further analyzed. ANOVA on RTs revealed a main effect of handle orientation (F (1,68) = 4.826, *p* = 0.031, ηp2 = 0.066): participants were slower at detecting right-handled stimuli (Log-RT: 6.34 ± 0.01) than left-handled stimuli (Log-RT: 6.32 ± 0.01). Furthermore, ANOVA results revealed a main effect of group (F (1, 68) = 3.384, *p* = 0.023, ηp2 = 0.13). Post hoc comparisons showed that RTs of participants assigned to the Grasp-with-barrier group (Log-RT: 6.4 ± 0.03) were slower than RTs of participants assigned to the Grasp-without-barrier group (Log-RT: 6.25 ± 0.01), no other comparisons were significant (See Fig. 3). The main effects were further qualified by a significant interaction (F (1, 68) = 5.37, *p* = 0.002, ηp2 = 0.192). Post hoc comparisons showed that RTs of participants assigned to the Grasp-without-barrier and Grasp-with-barrier groups were slower at detecting right-handled stimuli than left-handled stimuli (Grasp-without-barrier Group Log-RT: 6.27 ± 0.03 right-handled stimuli vs. 6.23 ± 0.03 left-handled stimuli, *p* = 0.002; Grasp-with-barrier Group Log-RT: 6.42 ± 0.03 right-handled stimuli vs, 6.38 ± 0.03 left-handled stimuli, *p* = 0.005). RTs of participants assigned to the Touch-without-barrier and Touch-with-barrier groups did not show any difference (Touch-without-barrier Group Log-RT: 6.32 ± 0.03 right-handled stimuli vs. 6.32 ± 0.03 left-handled stimuli, *p* = 0.957; Touch-without-barrier Group Log-RT: 6.34 ± 0.03 right-handled stimuli vs, 6.36 ± 0.03 left-handled stimuli, *p* = 0.103).

## Discussion

The aim of the present study was to investigate action–vision coupling by assessing whether and how making a visually presented object out of reach would impact on perceptual judgment. We, therefore, contrasted participants’ performance in judging the shape of a mug (i.e., it is being handled or not), which was sometimes presented behind a transparent barrier, immediately after motor training consisting of repeatedly executing either a reach-to-grasp or a reach-to-touch action without any visual feedback. There were two main findings.

The first finding was that participants were slower in judging the shape of the visually presented mug when it was right-handled compared to when it was left-handled, with this effect occurring immediately after the reach-to-grasp motor training only. This finding replicates a previous study from our group (Costantini et al. 2019), which showed that repeatedly performing a reach-to-grasp action induces a slowdown in visually judging a mug when its handle orientation was congruent with motor training. This slowdown has been hypothesized to be due to an aftereffect occurring between the motor training and the visual judgment. Participants were slower in the visual judgment task because their motor training with of the handled mug would decrease the strength of their motor processing of its handle in the congruent trials (Palumbo, D'Ascenzo, and Tommasi 2017).

The second main finding was that inserting a transparent barrier before the mug affects participants’ judgment performance, slowing down their reaction times compared to when the barrier was absent. How can this finding be explained? One might be tempted to appeal to a difference in visual acuity. On this view, the slowing down in perceptual judgment would be mainly accounted for by a decreased visual saliency when the barrier covered the mug. A further tempting explanation might attribute the barrier effect to a change of visual context. Indeed, there is evidence that the presence of unexpected items may interfere with the motor processing involved in object and action observation (Beauprez, Toussaint, and Bidet-Ildei 2018). Although we cannot fully rule out the possibility that differences in either visual acuity or visual context might have somehow impacted on participants’ reaction times, there are, however, good reasons to resist both temptations. First, participants were slower in judging the right-handled than the left-handled mug, even when it was visually presented behind the barrier. Second, the insertion of the barrier induced a slowdown in perceptual judgment after the reach-to-grasp motor training only. Indeed, no significant difference in reaction times was recorded when participants had to judge the handled mug either behind or without the barrier immediately after the reach-to-touch motor training.

Taking together, these data suggest an alternative hypothesis, which points to a barrier-specific modulation of the aftereffect induced by the motor training on perceptual judgment. According to this hypothesis, participants were slower in judging when the handled mug was visually presented behind the barrier because in this condition, the mug looked like something that could not to be acted upon, and this decreased the strength with which the representation of its handle was processed in virtue of interfering with the motor processes and representations which were recruited in their motor training and which would otherwise have speeded up their perceptual judgment.

Two different lines of evidence seem to jointly support this hypothesis. The first line of evidence comes from studies showing that repeatedly performing a certain action might induce a loss in function of visual perception of stimuli congruent with the motor training (Cattaneo et al. 2010; Musseler and Hommel 1997; Musseler, Steininger, and Wuhr 2001). In particular, Musseler and Hommel (Musseler and Hommel 1997) carried out a series of experiments in which participants were presented with masked left or right arrows shortly before executing an already prepared manual left or right key press. They found that the preparation of a spatially selective action (e.g. a left key press) went along with a temporary "blindness" to visual features with the same spatial attribute (e.g. a left pointing arrow). Furthermore, Cattaneo et al. (2010) asked participants to perceptually judge whether a hand perpendicularly touching a small ball was actually pushing or pulling it, after being motorically trained to perform blindfolded a push or a pull action. The results showed that after push training, participants were biased to judge the viewed perpendicularly touching hand as pulling the ball, while pull training had, as consequence, the opposite bias. Both biases vanished if a TMS pulse was delivered over participants’ ventral premotor cortex after their motor training, thus suggesting that the motor processes and representations recruited in the push–pull training were critically correlated to the aftereffect as measured by participant’s perceptual judgment.

A second line of evidence concerns how preventing from acting upon an object might interfere with the way in which features of this object are represented. For instance, Morgado et al. (Morgado, Gentaz, Guinet, Osiurak, and Palluel-Germain 2013) investigated whether the presence of a transparent barrier might impact distance perception. Their results showed that distances were perceived as being longer when the target object was grasped by reaching around a wide barrier. In addition, Costantini et al. (2010) asked participants to pantomime a reach-to-grasp action on the presentation of a right- or left-oriented handled mug. They found that participants were faster in responding with their reach-to-grasp pantomime when the handled mug was oriented congruently with the hand to be used. This effect was not found when the mug was presented beyond a plexiglas barrier.

It is worth noting that the present study involves three additional steps in comparison to Costantini et al. (2010). The first step consists in taking advantage of the barrier insertion to explore how action may impact on perception, while Costantini et al. explored how perceptions (e.g. the sight of a right/left-handled mug) affect action (e.g. the performance of a reach-to-grasp pantomime). The second step concerns the fact that the present paper extended the compatibility effect investigated by Costantini et al. (2010) to a categorization task, by showing that the barrier insertion impacted not only the lifting time in a reach-to-grasp pantomime but also the readiness to judge the perceived object. Finally, the third step is about the nature of the impact. While in Costantini et al. (2010), we found a facilitation effect; in the present study, the experimental paradigm was designed to test the possibility that the compatibility effect functions in the opposite direction, with the motor training making participants slower in categorizing the perceived object when this was oriented congruently with the trained hand.

On the basis of the two above-mentioned lines of evidence, we hypothesize that perceptual judgment might hinge, partially at least, on the same kind of processes and representations which are typically involved in planning and executing action. This would explain why repeatedly acting upon an object like a handled mug without any visual feedback might induce a perceptual aftereffect as measured by a slowing down in judgment performance when the handle of the mug was presented congruently with the trained hand. And this would also explain why perceptual judgment might be slowed down even more by the insertion of a transparent barrier before the handled mug, which prevented participants from acting upon it, thus interfering with their recruitment of related motor processes and representations.

Note that this hypothesis is compatible with an interpretation of ‘blindness to response-compatible stimuli’ in terms of a common coding of stimulus and response codes (Prinz 1990). According to this interpretation, a self-inhibition mechanism would prevent a common coding system from an endless perception–action cycle, by making it blind to response-produced effects. Such self-inhibition would affect the detection not only of the response-produced effects but also of the stimuli that resemble those effects and are temporarily close to the response (Musseler and Hommel 1997). This would explain why the perceptibility of response-compatible stimuli (e.g. right-handled mug) should be decreased immediately after the grasping training only. And it is possible that the insertion of the barrier impacted on the self-inhibition mechanism, by disrupting, partially at least, the action–perception coupling.

This seems also to shed light on the neuronal mechanism which allegedly underpins perceptual aftereffects induced by motor training. Perceptual aftereffects are typically associated with visual adaptation. There is much evidence that visual neuron responses can be selectively reduced by repeated exposure to specific visual stimuli, with systematic biases in perceptual tasks (Matsumiya and Shioiri 2014; Mohr, Rickmeyer, Hummel, Ernst, and Grabhorn 2016; Palumbo, et al. 2017). Cattaneo et al. (2010)’s findings indicated that these biases can be systematically induced by the adaptation of a specific class of sensorimotor neurons (e.g. mirror neurons), which selectively respond to both executed and observed actions. According to our hypothesis, the slowing down of participants’ performance in perceptual judgment could be due to a similar sensori-motor adaptation process.

It is well known that visual features of objects (e.g. the shape an handled mug) can be processed by a specific class of visuomotor neurons, that is, canonical neurons, which are typically recruited both when executing object-related actions, such as grasping, and when merely viewing objects with different sizes and shapes, without any intention to act on them (Jeannerod 1995; Jeannerod, Arbib, Rizzolatti, and Sakata 1995; Rizzolatti et al. 1988). Because these neurons are activated from both motor and visual inputs (Gerbella, Belmalih, Borra, Rozzi, and Luppino 2011; Matelli, Camarda, Glickstein, and Rizzolatti 1986), the effects of their firing history driven by repeatedly reaching-to-grasp a handled mug could be observed in the visual judgment on it. This would explain why participants were slower in their judgment performance when presented with a right-handled mug than with a left-handled mug, as their right hand only (not the left one) was involved in the reach-to-grasp training (Fig. 3).

Even more interestingly, a recent study on canonical neurons in monkeys demonstrated that their responses dramatically decrease when the target object looked like as something that could not be acted upon, being either too far from the viewer or behind a barrier (Bonini, Maranesi, Livi, Fogassi, and Rizzolatti, 2014). Similar results have been obtained in humans (Buccino, Sato, Cattaneo, Roda, and Riggio, 2009; Cardellicchio, Sinigaglia, and Costantini 2011, 2013). For instance, Cardellicchio et al. (2013) magnetically stimulated the primary motor cortex and recorded motor-evoked potentials (MEPs) from the right first dorsal interosseus (FDI) and opponens pollicis (OP), while participants were presented with a handled mug close either to them or to a virtual individual, such as an avatar. The results showed that MEPs dramatically decreased when the mug was presented behind a transparent barrier, thus appearing out of reach for both the participants and the avatar. Taken together, these data suggest that the insertion of a barrier before the target object might reduce the strength of the canonical neuron responses to it. The firing history of these neurons induced by motor training similar to that carried out in study would, therefore, be much less effective at the perceptual level. This would explain why the insertion of a transparent barrier before the handled mug selectively impacted participant’s performance, making them even slower in perceptually judging the mug.

Although further research is needed, our findings seem to enrich the view that visual perception can be affected by processes and representation involved in action preparation. Not only may acting on an object impact the way in which the object is perceptually judged, but also being prevented from acting on an object may also impact visual perception as measured by readiness to make perceptual judgements.

## Data Availability

Yes.
